# Fishing activity before closure, during closure, and after reopening of the Northeast Canyons and Seamounts Marine National Monument

**DOI:** 10.1038/s41598-021-03394-6

**Published:** 2022-01-18

**Authors:** John Lynham

**Affiliations:** Department of Economics, University of Hawai‘i at Mānoa, Honolulu, HI 96822 USA

**Keywords:** Environmental economics, Socioeconomic scenarios, Sustainability, Marine biology, Ecosystem ecology, Ecosystem services, Environmental economics, Population dynamics, Restoration ecology

## Abstract

Evaluation of the economic impacts of marine protected areas is hampered by the fact that it is impossible to observe what would have happened if the protected area had never been closed to fishing (the counterfactual). Catch reports and vessel tracks are used to perform an analysis of the potential negative economic impacts of establishing the Northeast Canyons and Seamounts Marine National Monument (located off the east coast of the United States of America) on three commercially important fisheries that were identified as having potential to be harmed. I conclude that there was little to no negative impact on any of the fisheries. I also test for, but find no evidence of, a Blue Paradox effect. Due to political factors largely unrelated to fisheries status, the protected area was reopened to commercial fishing on June 5th, 2020. I use this event, which was reversed sixteen months later, to test whether there were any economic benefits from reopening. I do not observe an increase in catch, a reduction in distance traveled, or an increase in relative fishing effort inside the protected area (compared to historical trends), consistent with the post-closure findings.

## Introduction

Marine protected areas (MPAs) are being implemented across the world as tools for conserving nature, enhancing marine biodiversity, sequestering carbon in undisturbed sea bottoms, mitigating the effects of climate change, and for promoting sustainable fisheries^1–5^. The results to date on how MPAs affect adjacent fisheries are mixed and this remains an area of active debate, both theoretically and empirically^6–16^. MPAs benefit fish species protected within their boundaries (especially less migratory species) and this may lead to future spillover benefits for fisheries^17,18^. But, in the short term, fishers are typically displaced from areas they have fished in (sometimes for long periods of time) and this may cause economic harm^19,20^. These types of impacts are often raised by fishing industry interests to challenge MPA designations.

**Figure 1:**
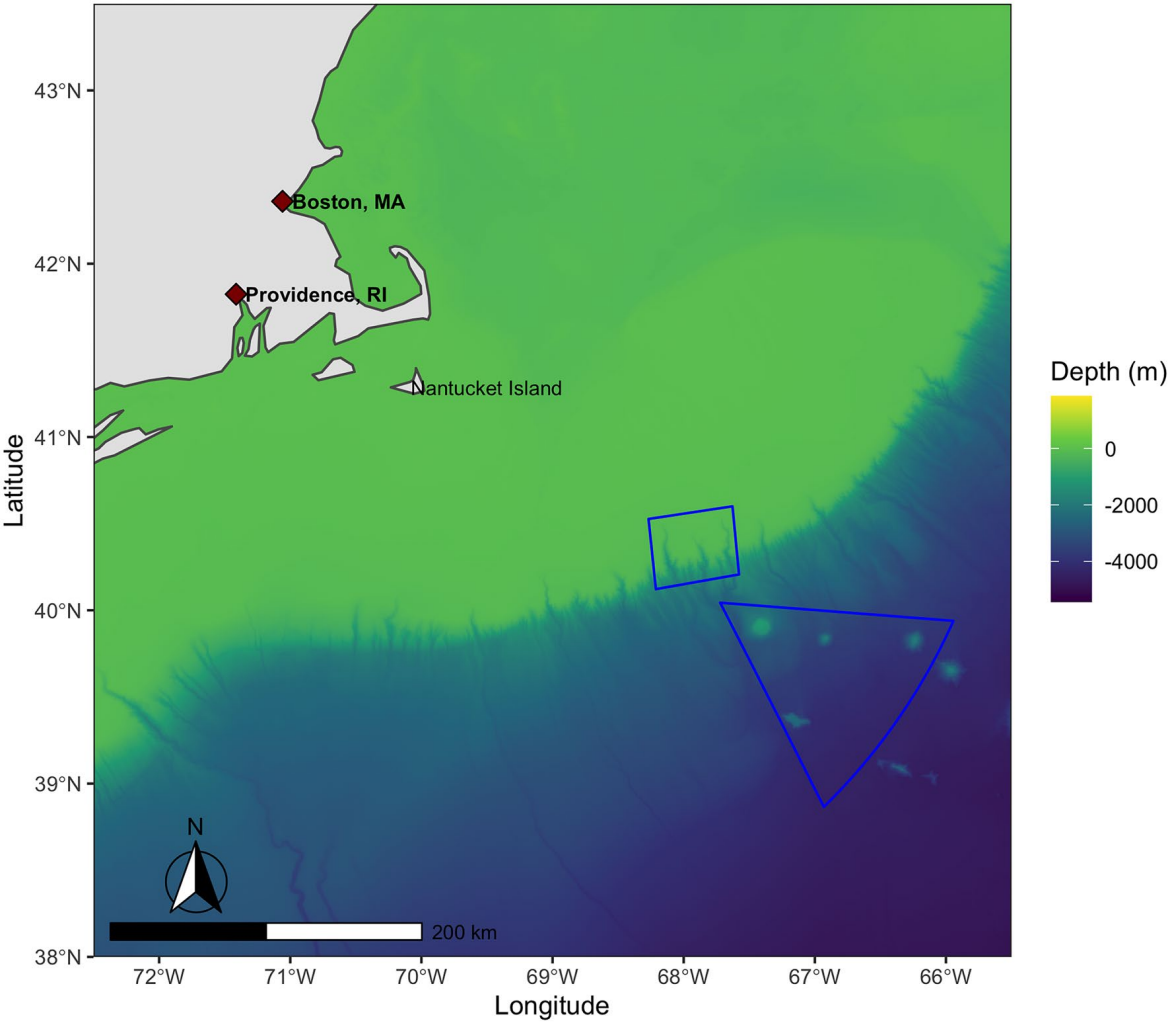
Northeast Canyons and Seamounts Marine National Monument. The monument boundaries are shown in blue, along with the nearest landmass, two nearest cities, and bathymetric depth. Map drawn by the author using R version 4.1.1 and RStudio 1.4.1717. Bathymetry data was obtained from the General Bathymetric Chart of the Oceans.

The Northeast Canyons and Seamounts Marine National Monument is a highly protected MPA off the east coast of the United States of America that was created by US President Barack Obama on September 15, 2016. It is the only national monument in US Atlantic waters and is comprised of two separate areas—a Canyons Unit and a Seamounts Unit—situated approximately 170 km southeast of Nantucket Island in the state of Massachusetts (Fig. 1). The 12,275 sq km monument—an area slightly larger than the island of Jamaica—protects a diverse array of species, including an abundance of marine mammals and sensitive, long-lived deep-sea corals^21,22^.

In order to achieve the monument’s conservation objectives, most forms of commercial fishing were banned within the monument’s boundaries shortly after its creation, with the remaining fisheries (lobster and red crab) to be phased out over a 7 year time period. This contrasts with the US national monuments located in the Pacific Ocean, where all fishing was permanently banned upon their creation^23^. Furthermore, the ban on commercial fishing did not come into effect at the moment of the monument’s creation but was delayed by 2 months until November 14, 2016. This meant that there was a time period when fishers knew they would soon be excluded from fishing in the monument but could still fish there. This allows for a test of the “Blue Paradox” hypothesis: anticipation of a marine reserve catalyzes a race-to-fish that undermines the intended conservation objectives of the reserve^24–26^. This is relevant for US, EU, and global efforts to increase the amount of ocean territory protected within MPAs^2,27,28^. For example, it has been argued that if MPA announcements were to trigger preemptive fishing, this could temporarily increase the share of over-extracted fisheries globally from 65 to 72%^24^. The local fishing industry has argued that the monument’s closure to most forms of commercial fishing caused significant financial hardship, with claims that as many as 80 vessels used to fish in the monument^29^. These claims are consistent with arguments more generally heard from fishing interests that commercial fishing prohibitions in U.S. ocean areas to protect biodiversity have reduced yield and inflicted economic harm^30^. Citing economic harm, US President Donald Trump reopened the monument to all legally permitted fishing operations on June 5, 2020. On October 8, 2021, President Joseph Biden reversed this action by restoring the 2016 protections.

I analyze these claims of economic harm using three primary data sources on catch and vessel movements (see “Methods” section). I focus on the three fisheries indicated by data from fishery management bodies [namely the New England Fishery Management Council and the National Oceanic and Atmospheric Administration (NOAA)] as the most significant fisheries with the potential to be adversely affected by the closures that followed monument designation^31^. The first is the squid/butterfish trawl fishery, which primarily targets longfin (also known as loligo) squid (*Doryteuthis (Amerigo) pealeii*). The longfin squid fishery generally consists of an offshore fishery in the boreal winter months (when squid, together with butterfish (*Peprilus triacanthus*), congregate offshore along the continental shelf break) and an inshore fishery in the late spring through early autumn months. For the purposes of this analysis, I define the offshore squid season as September to March. The second is the Atlantic mackerel (*Scomber scombrus*) trawl fishery for which the fishing season is primarily in the winter (landings are typically reported from November until mid-May, with most landings reported between January and March). I define the mackerel season as November to March. The third is the Atlantic tuna/swordfish longline fishery, which targets multiple tuna species and swordfish (*Xiphias gladius*), and operates year-round. An Atlantic Tuna Longline Commercial Fishing Permit gives the holder to right to land North Atlantic Albacore Tuna (*Thunnus alalunga*), Atlantic Skipjack Tuna (*Katsuwonus pelamis*), Atlantic Bigeye Tuna (*Thunnus obesus*), Western Atlantic Bluefin Tuna (*Thunnus thynnus*), and Atlantic Yellowfin Tuna (*Thunnus albacares*).

It is challenging to identify the causal impact of a marine protected area because it is usually impossible to observe what would have happened if the protected area had never been closed to fishing (the counterfactual)^9,32^. What is unique about this particular setting is that I am able to observe fishing activity before the announcement of an MPA, after the announcement but before closure, after closure, and after re-opening. To date there has been no empirical evaluation of how a commercial fishery is impacted by suddenly regaining access to a “permanently” closed area. Although some industry participants may have expected that the monument might be reopened to fishing, the exact timing was sudden and could not have been easily predicted, thus satisfying the conditions for a “natural experiment”^33^. The re-opening provides a second opportunity to test how the protected area impacts fishing activity.

I have four main findings. First, only a small fraction (less than 1%) of historical fishing grounds for the fisheries analyzed were lost due to the monument. More than 99% of the locations that vessels used before the monument was created were still open to fishing after it was created. Second, catch does not appear to have declined in the three fisheries following the creation of the monument. Pounds landed have not gone down for squid, butterfish, mackerel, and tuna/swordfish in correlation with the fishing prohibition, especially when compared to adjacent regions with landings for the same species. Third, none of the potentially impacted fleets were forced to travel further to fish after the monument was closed to fishing. Fourth, the absence of impacts of closing the monument are mirrored by the absence of impacts of re-opening the monument. I do not observe an increase in catch, a reduction in distance traveled, or an increase in relative fishing effort inside the monument (compared to historical trends) for any of the fisheries. On average, since reopening, 99% or more of fishing activity is still taking place outside the monument. The economic arguments made against the 2016 commercial fishing prohibition and in favor of the 2020 re-opening do not appear to be supported by data on landings and vessel movements.

## Results

### Lost Fishing Grounds

**Figure 2:**
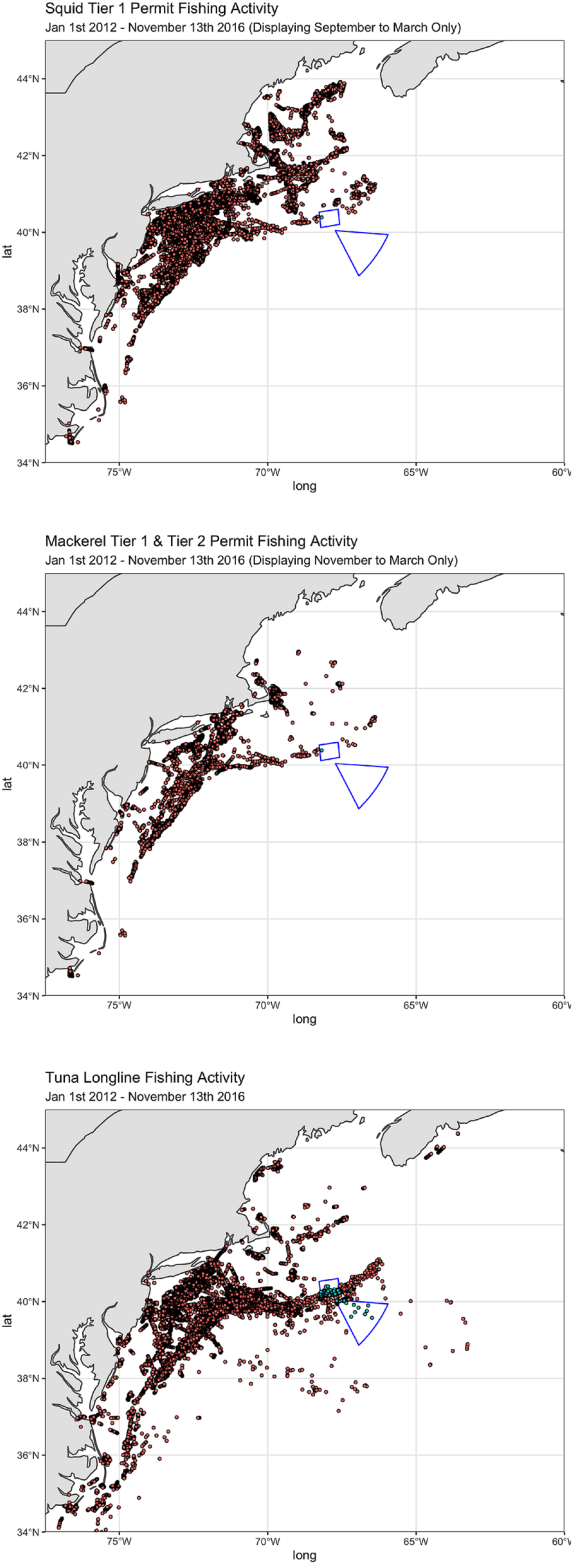
Fishing Locations Prior to Closure by Fishery. Red dots indicate activity outside the monument, light blue dots indicate activity inside the monument. The monument boundaries are shown in blue. Maps drawn by the author using R version 4.1.1 and RStudio 1.4.1717.

I start by estimating how much activity took place inside the monument before it was closed. I differentiate between fishing activity and all vessel activity (see “Methods” section). In Fig. 2, I display a visual summary of fishing activity for each fishery from 2012 to 2016, prior to the closure of the monument. Observed vessel locations are color-coded by whether they occur inside or outside of the monument. The maps show the full extent of all fishing activity (except for a few trips by tuna longline vessels to extremely distant locations, e.g. the Pacific Ocean). The implication is fairly clear: very little historical fishing effort took place inside the monument for any of the three fisheries. The importance of the continental shelf break and slope to the longline fishery can be clearly seen in the bottom panel of Fig. 2.

Table 1 provides a more complete summary of these results. For all three fisheries, it can be seen that more than 99% of both fishing activity and all vessel activity was taking place outside the monument even when the monument was open to fishing. It is particularly extreme for the squid/butterfish and mackerel vessels: over 99.99% of vessel and fishing activity took place outside the monument when the monument was still open to fishing for these species. Based on these initial results, it seems unlikely that the closure of the monument would impact either of the three fisheries in any meaningful way. The most likely candidate to experience a negative impact is the tuna longline fishery, which appears to have had slightly more relative fishing effort inside the boundaries of the monument before it was closed.

**Table 1:** Percentage of Vessel activity and fishing activity outside the monument.

	% of vessel activity	% of fishing activity	% of vessel activity	% of fishing activity
	Outside monument	Outside monument	Outside monument	Outside monument
	Prior to closure	Prior to closure	Following reopening	Following reopening
Squid vessels	99.999	99.998	99.997	99.994
Mackerel vessels	99.998	99.992	99.997	100
Tuna vessels	99.931	99.445	99.978	99.553

#### Blue Paradox?

**Figure 3:**
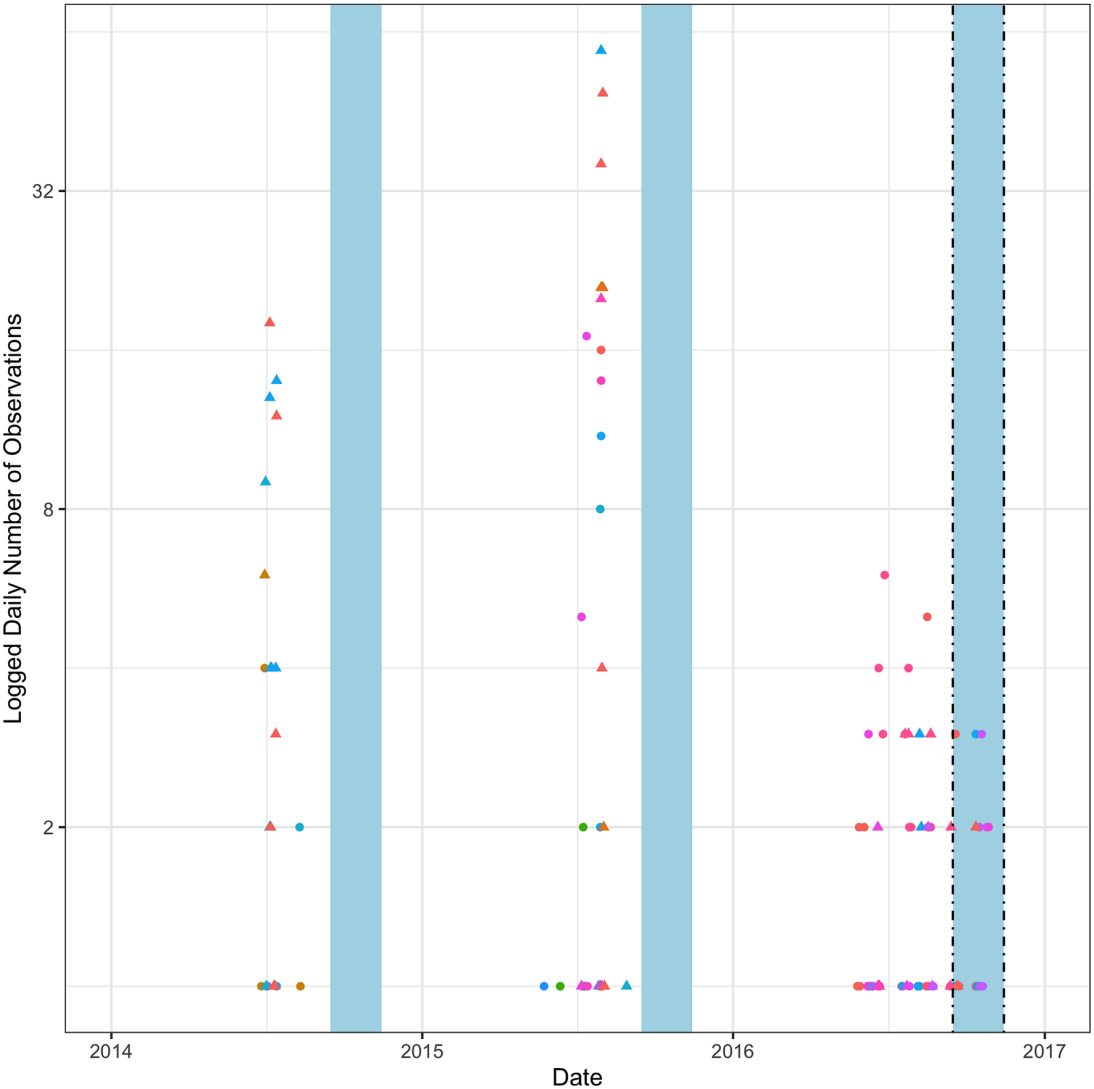
Tuna blue paradox. This figure displays a scatter plot of the number of times a vessel is recorded by GFW as being within the boundaries of the monument. Different vessels are displayed with different colors. Observations are grouped by whether GFW categorized them as fishing events (triangles) or non-fishing events (circles). The blue shaded regions represent the same calendar dates in each year (September 15th to November 14th). The dashed vertical lines indicate the announcement of the monument (September 15th, 2016) and its subsequent closure to tuna fishing (November 14th, 2016). The vertical axis is logged to the base two.

**Figure 4:**
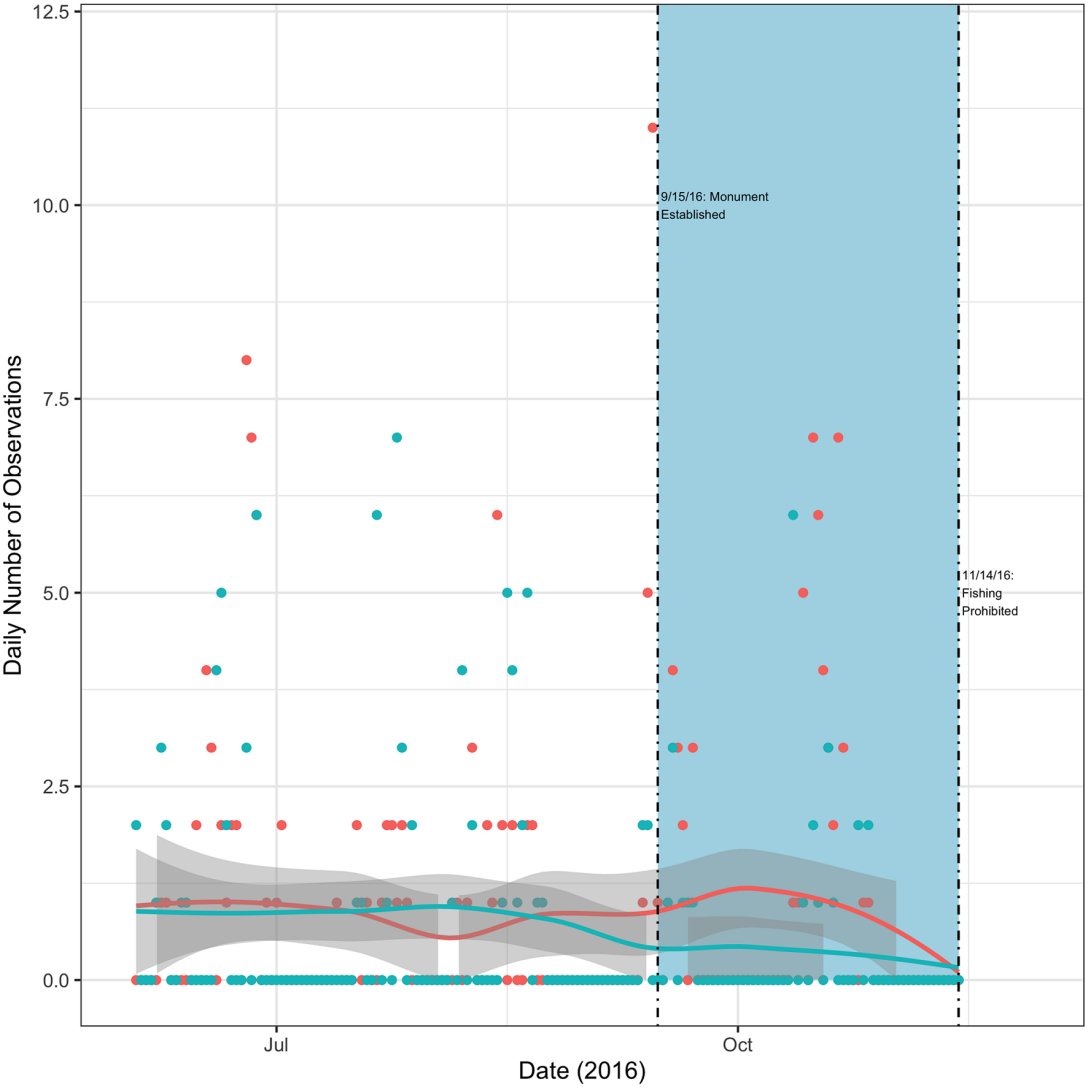
Total vessel activity inside and near the monument. This figure displays a scatter plot of the total number of daily observations for tuna vessels either inside or outside but near the monument, along with their respective LOESS regression lines. Near the monument is defined as occurring within a box with bounding coordinates of ($40.6^{\circ }$, $-\,68.268^{\circ }$) and ($38.865^{\circ }$, $-\,65.943^{\circ }$). These coordinates correspond to the most northern, most western, most southern, and most eastern coordinates within the monument itself (coordinates provided by NOAA). Total observations inside the monument are color-coded blue and total observations outside but near are color-coded red. The dashed vertical lines indicate the announcement of the monument (September 15th, 2016) and its subsequent closure to tuna fishing (November 14th, 2016). The blue shaded region indicates the time period over which I might expect a Blue Paradox effect.

For tuna vessels, I initially appear to observe a small Blue Paradox prior to closure of the monument (Fig. 3). Following the announcement of the monument closure but before longline vessels were legally excluded, a number of vessels show up inside the monument and some of this activity is categorized as fishing. This initially seems unusual since there was very little recorded longline fishing in or near the monument at this time of the year in previous years. In Fig. 3, the same time period is shaded in light blue for 2014 and 2015 and it can be observed that no vessels fished in the monument area at this time of year in previous years. However, upon closer inspection there appears to be a much simpler explanation for the apparent ramp-up in effort following the announcement of the protected area: fishing conditions were good in this general area in the fall of 2016. This is shown in Fig. 4 and confirmed by regression tests in Table 2: effort inside the monument post-announcement is matched by effort near but outside the monument. I performed a similar analysis for the squid/butterfish and mackerel fisheries but do not share the results here since they are trivial. The 2 months period between announcement and closure does not overlap with the main squid and mackerel fishing seasons in the vicinity of the monument so there was no observed vessel activity inside the monument during this time period. In a way, this could be considered further evidence against the Blue Paradox, as the announcement did not trigger additional fishing activity that would normally be unprofitable.

**Table 2:** Blue paradox hypothesis tests.

	Dependent variable			
	Total daily observations		Inside the monument	
	OLS	Poisson	OLS	Probit
	(1)	(2)	(3)	(4)
Intercept	0.913***	− 0.091	0.002***	− 2.898***
	(0.158)	(0.196)	(0.0003)	(0.064)
Inside monument	− 0.125	− 0.147		
	(0.224)	(0.275)		
After the announcement	− 0.012	− 0.013	− 0.0001	− 0.0004
	(0.261)	(0.319)	(0.0002)	(0.074)
Inside * After	− 0.367	− 0.641		
	(0.369)	(0.489)		
Vessel dummies	No	No	Included	Included
Observations	330	330	117,905	117,905
$\hbox {R}^{2}$	0.013		0.002	

For both regressions, the sample is restricted to observations between January 1st and November 14th, 2016. For the regressions in Columns (3) and (4), the sample of vessels is restricted to vessels with at least one record of activity inside the monument. Standard errors presented in parentheses. The regressions in Columns (1) and (3) are estimated using Ordinary Least Squares and standard errors have not been adjusted. Column (2) is etimated using a Poisson regression and standard errors are clustered at the inside/outside level. Column (4) is estimated using a probit model and standard errors are clustered at the vessel level. Asterisks indicate the results of individual two-sided tests of statistical significance: *${p}<0.1$; **$p<0.05$; ***$p<0.01$.

### Catch

**Figure 5:**
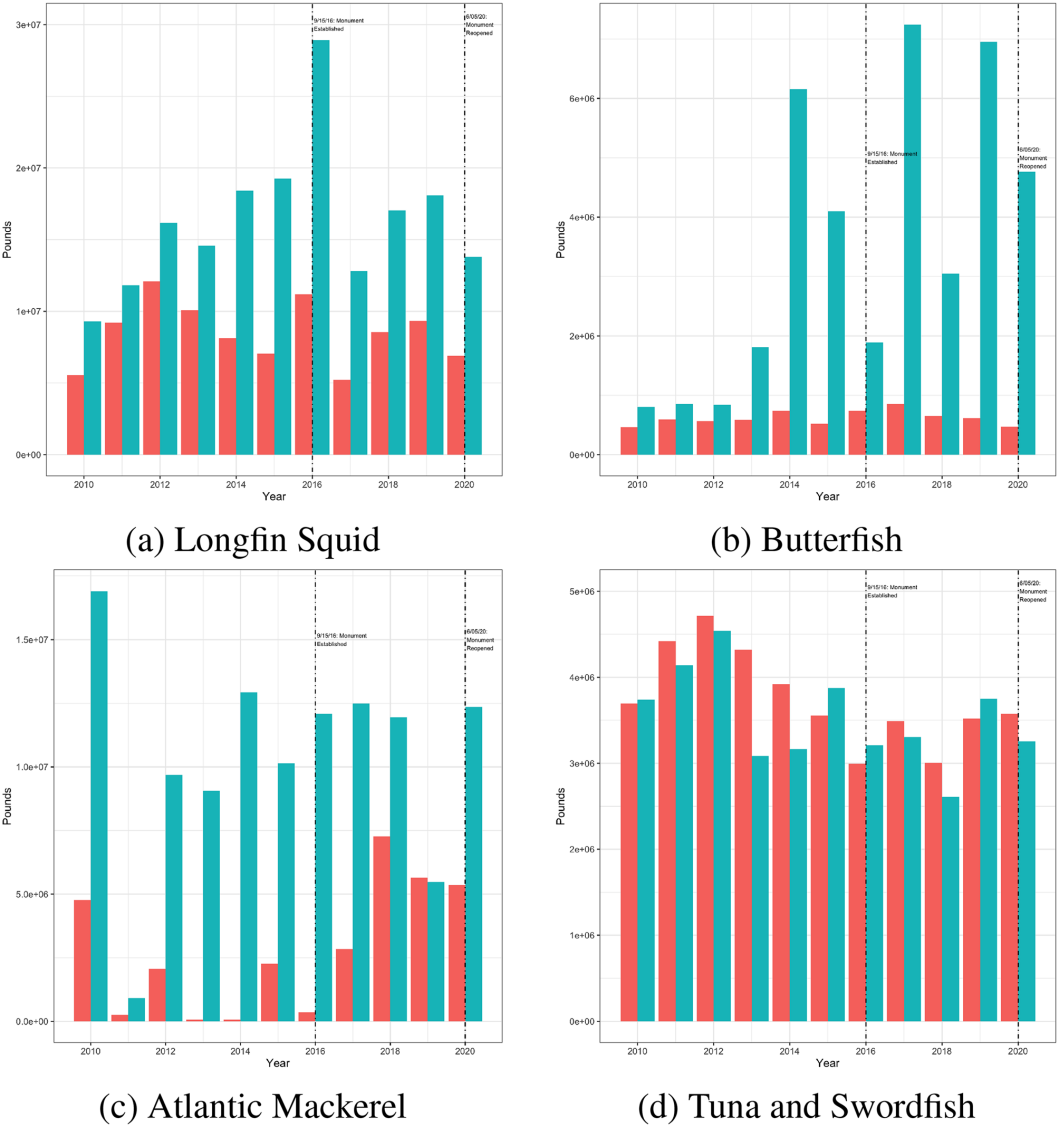
Panels (**a**)–(**c**) Landings by Year in the Mid-Atlantic (red) and North Atlantic (blue) Regions. Panel (**d**): Landings by Year in the South Atlantic Region (red) and Mid− and North Atlantic Regions combined (blue).

**Figure 6:**
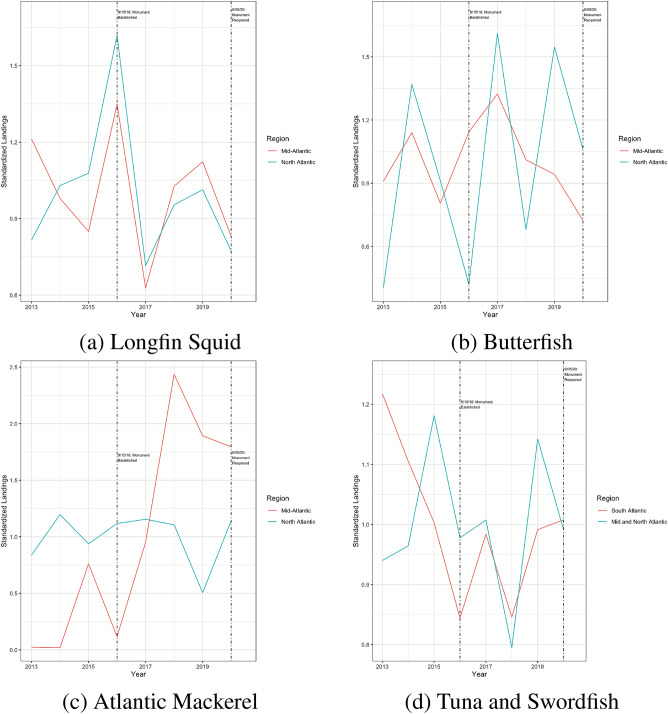
Panels (**a**)–(**c**) Standardized landings by Year in the Mid-Atlantic (red) and North Atlantic (blue) Regions. Panel (**d**): Standardized landings by Year in the South Atlantic Region (red) and Mid− and North Atlantic Regions combined (blue).

In Fig. 5, I summarize each of the three fisheries’ annual landings for two different regions: (1) likely to be impacted and (2) unlikely to be impacted. Since the monument is located offshore of a number of northeastern US states, it makes sense generally to assume that any disruption to catch would be observed primarily in the North Atlantic region (the coastal states of Maine, New Hampshire, Massachusetts, Rhode Island, and Connecticut) rather than the Mid-Atlantic (the coastal states of New York, New Jersey, Delaware, Maryland, and Virginia). Therefore, for the squid/butterfish and mackerel fisheries, I define the North Atlantic as the impacted region and the Mid-Atlantic as the control region. This is supported by documents prepared by the New England Fishery Management Council^31^. This also aligns with the data on vessel activity inside the monument before it was closed: the majority of squid and mackerel vessels who fished in the monument were registered to addresses in the North Atlantic region. For the tuna longline fishery, I extend the potentially impacted region to include the Mid-Atlantic and use the South Atlantic (the coastal states of North Carolina, South Carolina, Georgia, and Florida) as the control region for two reasons. First, the longline fleet tends to be more wide-ranging than the other two fisheries. Second, a number of longline vessels with registered addresses in the Mid-Atlantic region fished in the monument before it closed. Figure 6 presents the same data except standardized by dividing each time series by its mean value for 2013–2020.

The overall message from Figs. 5 and 6 is fairly clear. Landings vary from year to year but there is no noticeable drop in landings following the closure of the monument, especially when compared to adjacent regions targeting the same species. The only species that appears to exhibit a differential trend between impacted and control post-closure is mackerel but this is most likely due to the lower and highly variable landings in the mid-Atlantic region for mackerel (Panel (c) of Fig. 5). This is also the fishery with the least amount of fishing effort inside the monument pre-closure so it would be surprising if this was the only fishery to experience a decline in landings as a result of the monument area’s closure. Likewise, the only fishery to report a noticeable increase in landings post-reopening is mackerel and most of this would have been caught prior to June 5, 2020. In Table 3, I present a summary of regression tests of whether landings increased or decreased in the impacted regions following closure. I do not observe any statistical evidence of a drop in landings. In fact, if anything, I observe weak evidence that butterfish landings increased in the North Atlantic (relative to the mid Atlantic) following the closure of the monument.

**Table 3:** Landings regressions.

	Squid	Butterfish	Mackerel	Tunas and swordfish
	(1)	(2)	(3)	(4)
Intercept term	15.987***	13.295***	13.126***	15.178***
	(0.116)	(0.204)	(0.473)	(0.057)
Impacted group	0.600***	1.079***	2.772***	− 0.070
	(0.165)	(0.289)	(0.669)	(0.080)
Post monument	− 0.161	0.163	2.275**	− 0.160
	(0.212)	(0.373)	(0.864)	(0.103)
Treatment effect	0.151	0.957*	− 2.122	0.026
	(0.300)	(0.528)	(1.222)	(0.146)
Observations	20	20	20	20
$\hbox {R}^{2}$	0.585	0.720	0.573	0.236

The dependent variable in all regressions is the log of pounds landed but each column is for a separate fishery. Each regression tests whether pounds landed decreases following the monument closure in states near the monument, relative to states further away. The sample runs from 2010 to 2020. Standard errors presented in parentheses. Asterisks indicate the results of individual two-sided tests of statistical significance: *$p<0.1$; **$p<0.05$; ***$p<0.01$.

**Figure 7:**
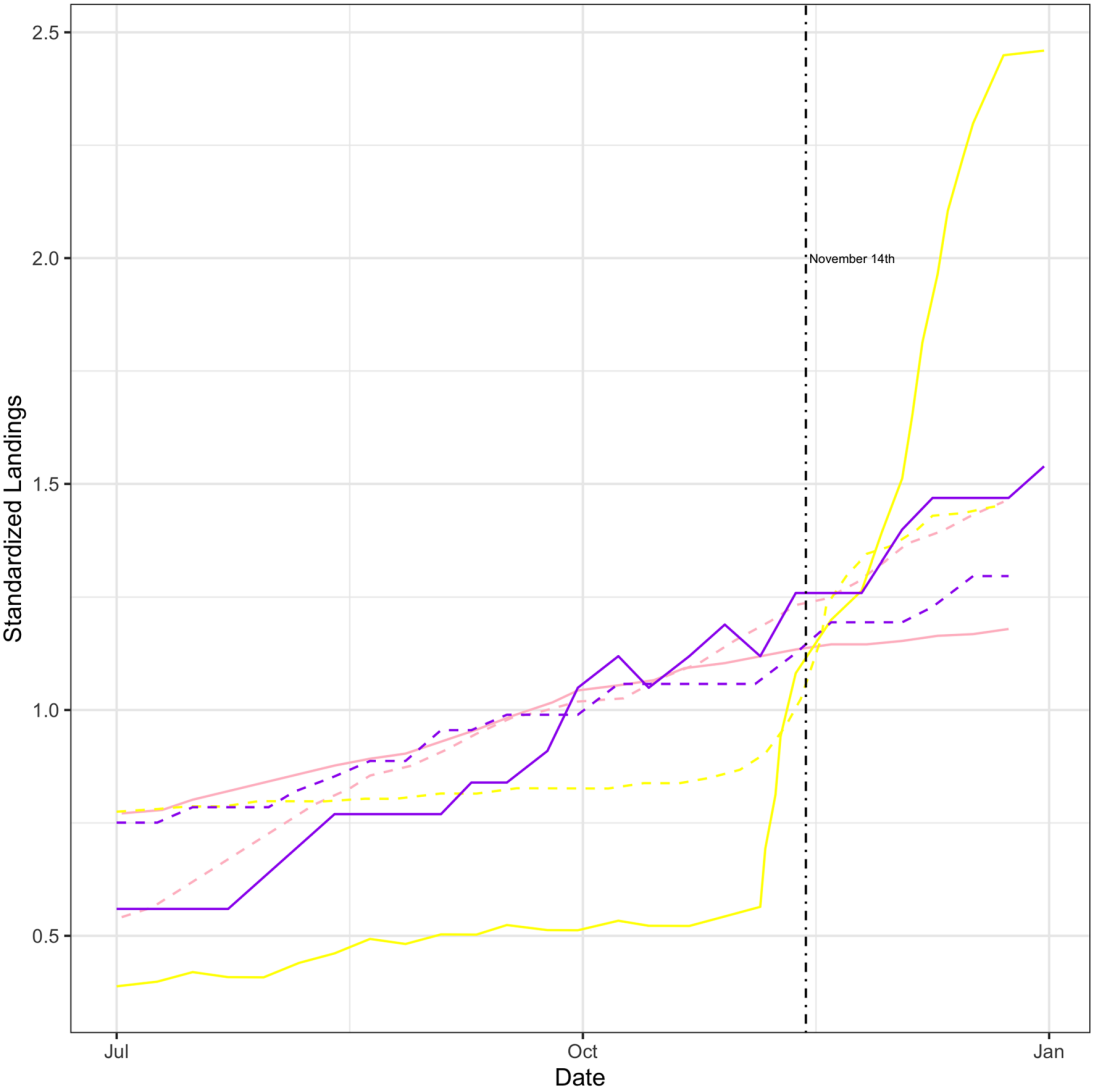
Standardized weekly landings for the North Atlantic and Mid-Atlantic regions combined. Pink indicates squid, yellow indicates mackerel, and purple indicates butterfish. Solid lines are landings by week for 2016 and dashed lines are landings by week for 2015. The vertical line indicates the date of the monument closure in 2016.

As an additional test, in Fig. 7, weekly cumulative landings are plotted for squid, mackerel, and butterfish (weekly quota monitoring reports are not available for tuna species). These landings are aggregated across the North Atlantic and Mid-Atlantic region and landings are standardized by dividing by mean landings for the weeks shown. The lines in Fig. 7 are color-coded by species and different line types indicate landings for either 2015 or 2016. It can be seen that there is no abrupt step down in catch for any species at the time of closure of the monument on November 14th, 2016. Furthermore, the trends of landings pre− and post-closure for all species follow the same trends as in 2015. For example, none of the fisheries exhibit consistently lower catches following closure in 2016, compared to what would be expected based on the prior year’s landings.

### Distance traveled

**Figure 8:**
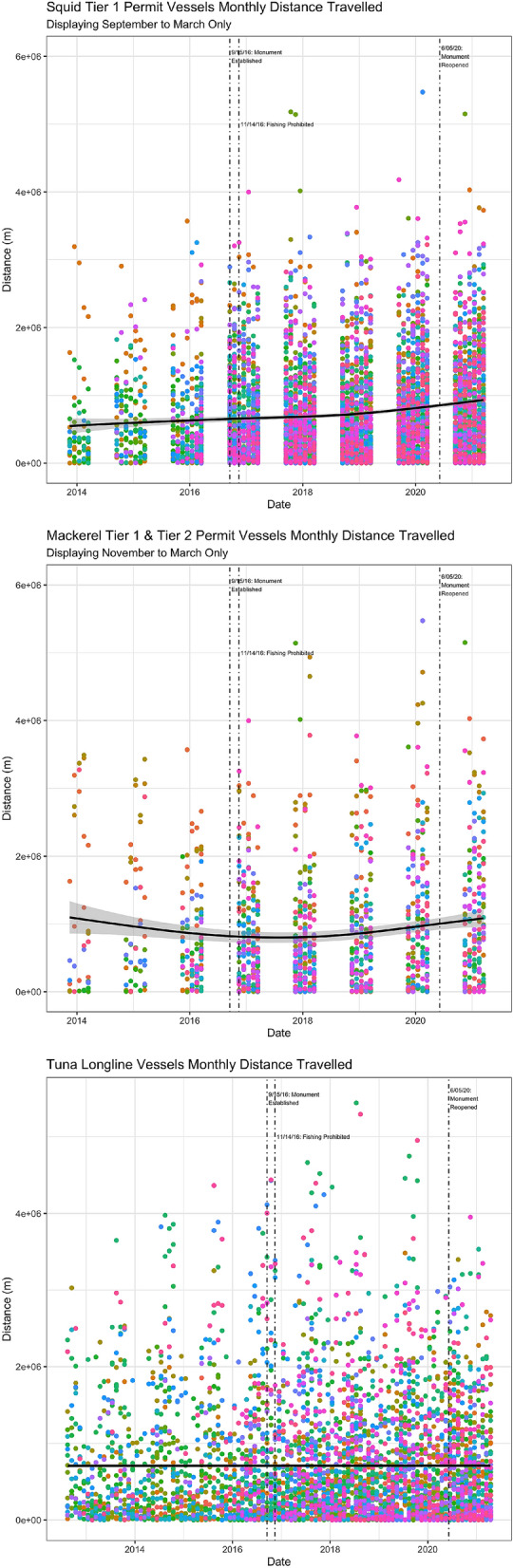
Monthly Distance Traveled by Fishery. Different colors indicate different vessels. Locally estimated scatterplot smoothing (LOESS) regression line displayed in black. Figures generated by the author using R version 4.1.1 and RStudio 1.4.1717.

Even if landings have not declined as a result of the monument closure, the monument could be affecting profitability by inconveniencing vessels and causing them to travel further or cover more ground because they cannot fish within the monument (this would be reflected as a drop in catch per unit effort, where effort is measured in terms of distance traveled or hours at sea). To test for this type of effect, I use the vessel movement data to calculate monthly distance traveled by each vessel identified in each fishery. See Fig. 8 for a summary of distance traveled by fishery. Visually there is no evidence of an abrupt increase in distance traveled following the closure of the monument and no evidence of a decrease in distance traveled following re-opening. In Table 4, I present a summary of regression tests of whether there was an increase in monthly distance traveled when the monument was closed. I do not observe any statistical evidence of an increase in distance traveled. In fact, if anything, I observe weak evidence that distance traveled decreased in the squid fishery following the closure of the monument.

**Table 4:** Distance Regressions.

	Squid	Mackerel	Tuna
	(1)	(2)	(3)
Monument closed	− 0.087*	0.094	0.026
	(0.045)	(0.100)	(0.053)
Vessel dummies	Included	Included	Included
Observations	4983	1202	4076
$\hbox {R}^{2}$	0.255	0.325	0.174

The dependent variable in all regressions is the log of monthly distance traveled. Each column corresponds to a different fishery. The sample runs from 2012 to 2021. Standard errors clustered at the vessel level presented in parentheses. Asterisks indicate the results of individual two-sided tests of statistical significance: *$p<0.1$; **$p<0.05$; ***$p<0.01$.

### Reopening

**Figure 9:**
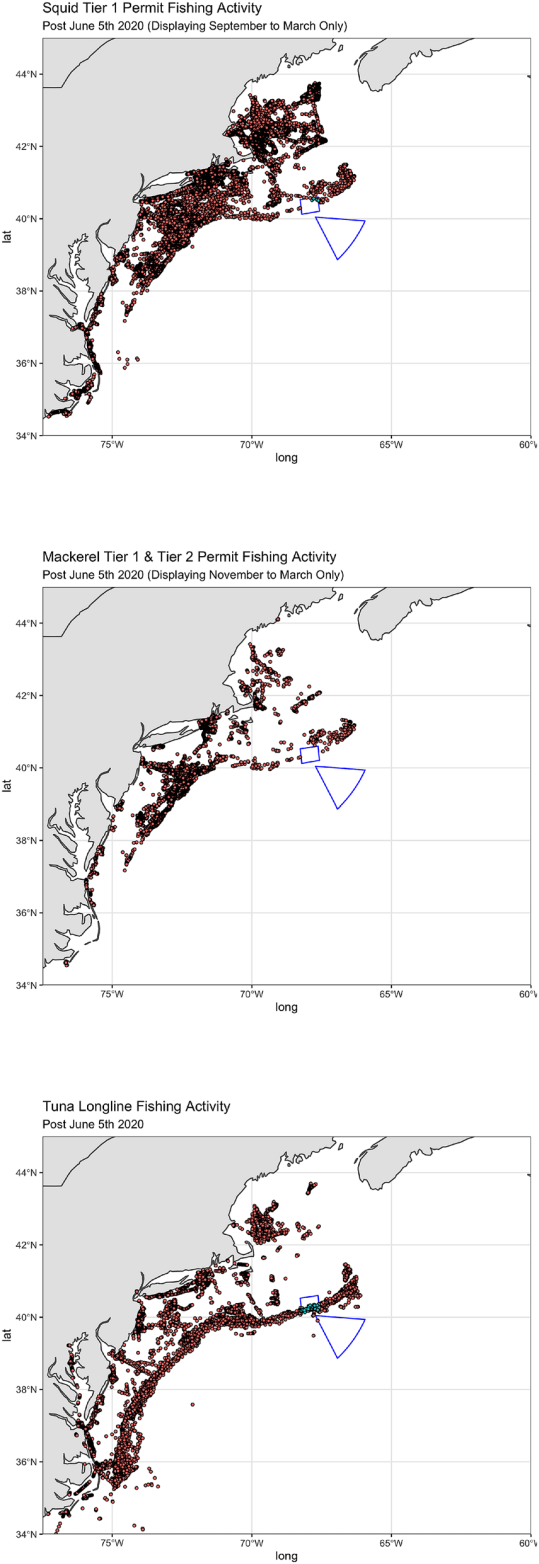
Fishing Locations After Reopening by Fishery. Red dots indicate activity outside the monument, light blue dots indicate activity inside the monument. The monument boundaries are shown in blue. Maps drawn by the author using R version 4.1.1 and RStudio 1.4.1717.

In Fig. 9, I display a visual summary of vessel activity following the reopening of the monument on June 5, 2020. Observed vessel locations are again color-coded by whether they occur inside or outside of the monument. The pattern is almost identical to before the monument closed: very little vessel activity in relative terms took place inside the monument. Table 1 confirms that, following reopening, 99% or more of fishing activity still took place outside the monument. In fact, not a single mackerel vessel was observed fishing inside the monument in the 11 months following its reopening.

**Figure 10:**
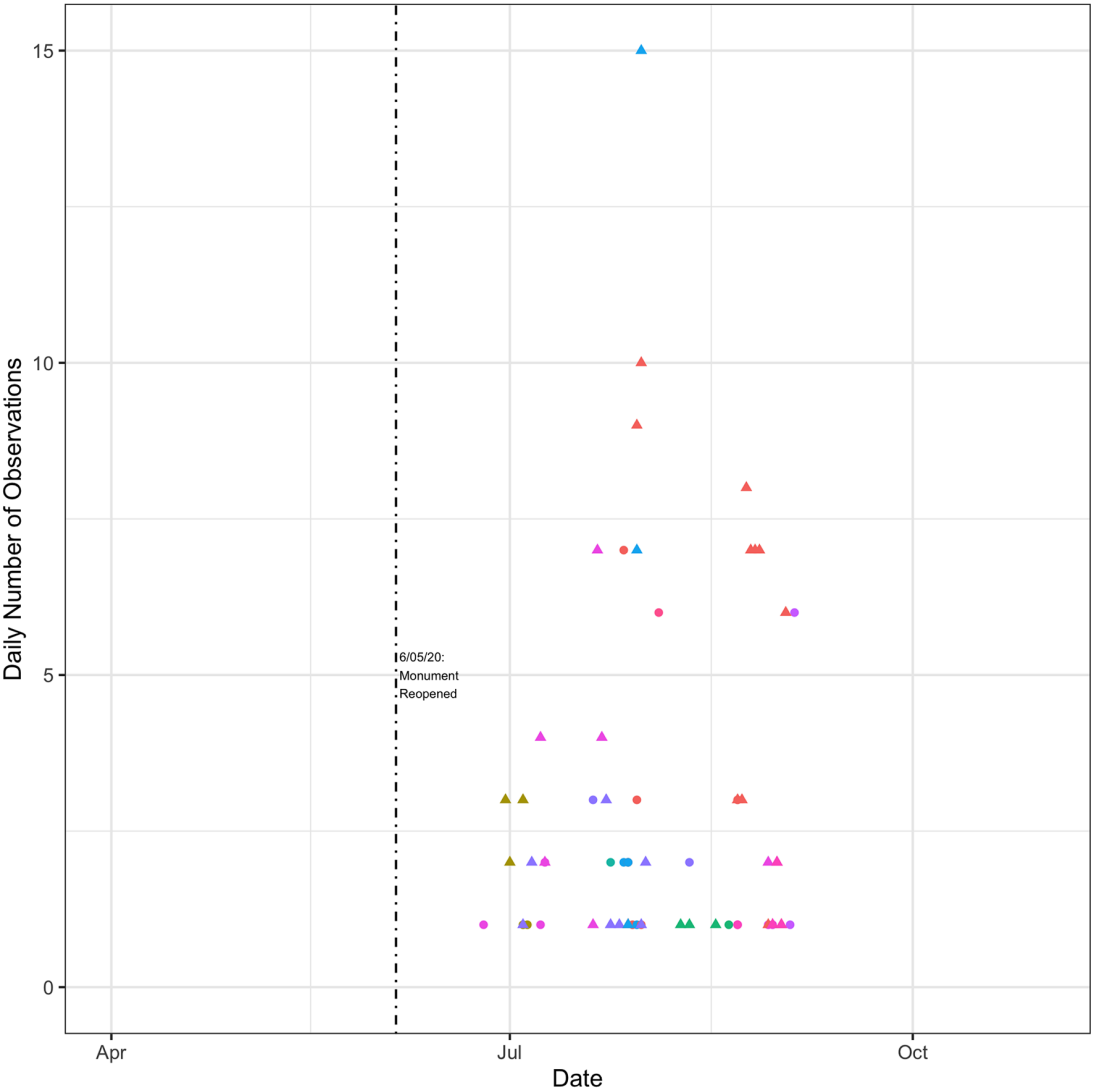
Tuna Vessel Activity Inside the Monument Post-Reopening. This figure displays a scatter plot of the number of times a vessel is recorded by GFW as being within the boundaries of the monument. Different vessels are displayed with different colors. Observations are grouped by whether GFW categorized them as fishing events (triangles) or non-fishing events (circles). The dashed vertical line indicates the re-opening of the monument. Days with values of zero are omitted from the graph.

**Figure 11:**
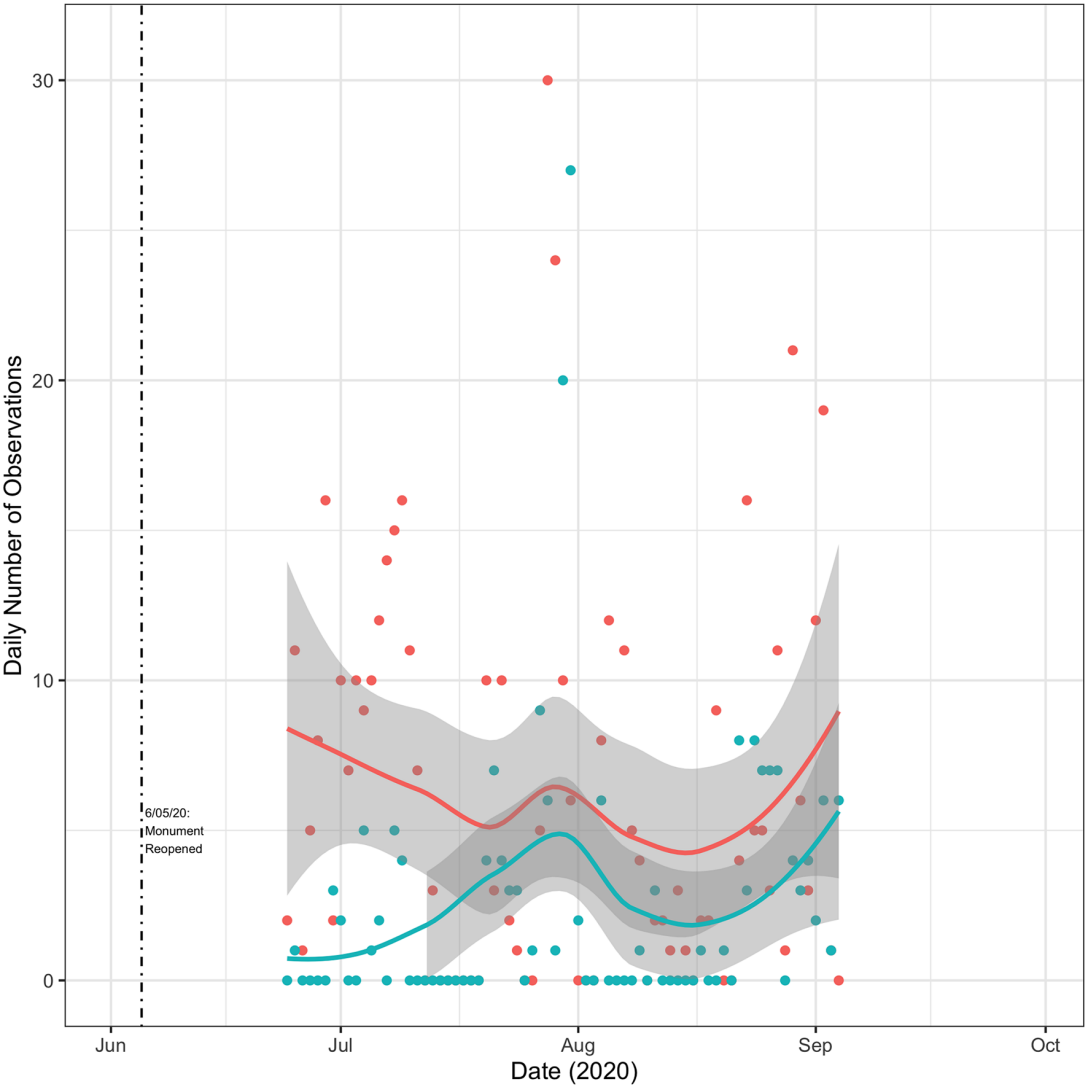
Tuna vessel activity inside and near the monument post-reopening. Blue dots indicate the total number of daily observations inside the monument. Red dots indicate the total number of daily observations outside but near the monument. Near the monument is defined as occurring within a box with bounding coordinates of ($40.6^{\circ }$, $-\,68.268^{\circ }$) and ($38.865^{\circ }$, $-\,65.943^{\circ }$). These coordinates correspond to the most northern, most western, most southern, and most eastern coordinates within the monument itself (coordinates provided by NOAA). Days with values of zero for both categories are omitted from the graph. Figures generated by the author using R version 4.1.1 and RStudio 1.4.1717.

**Figure 12:**
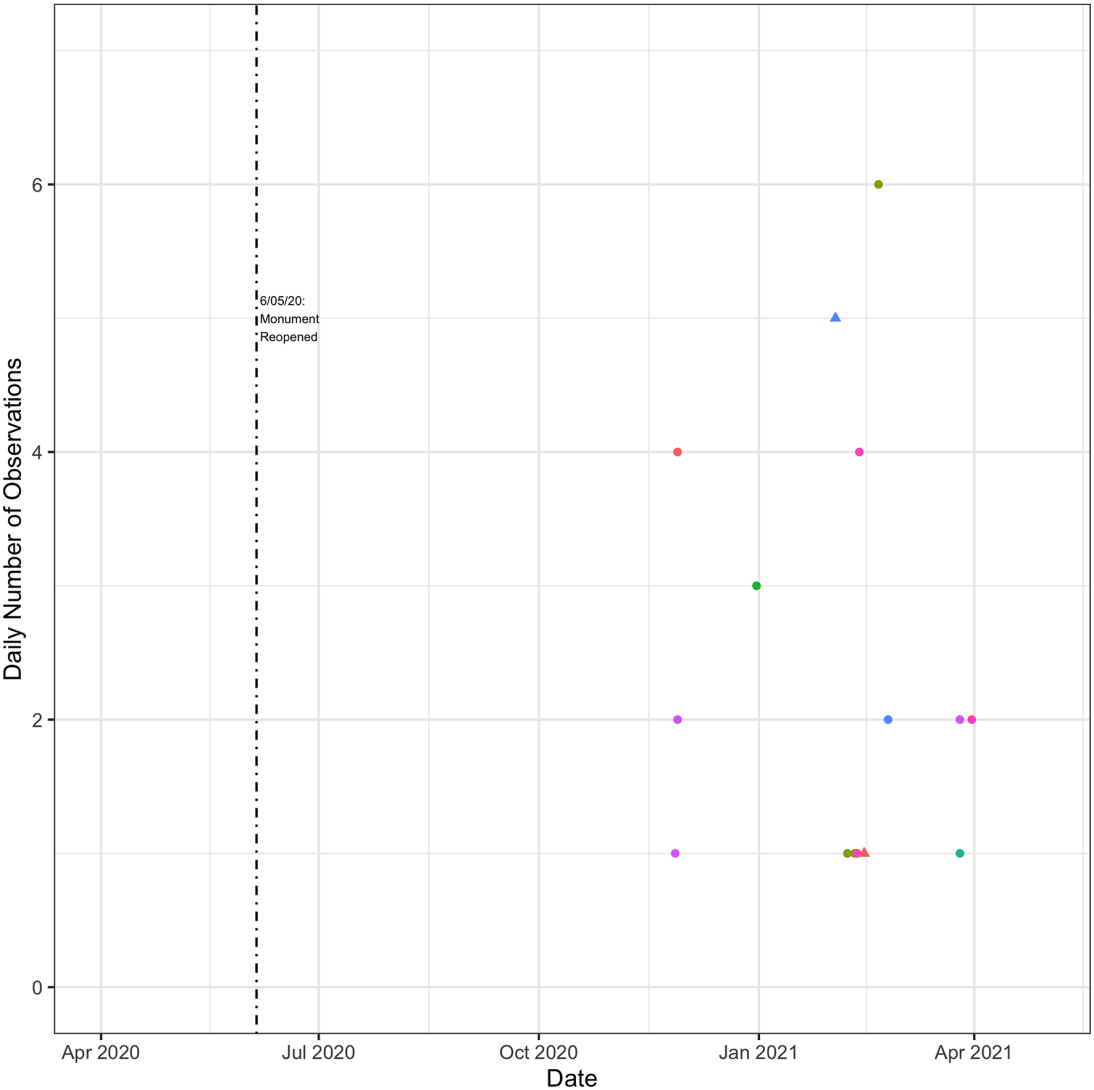
Squid vessel activity inside the monument post-reopening. this figure displays a scatter plot of the number of times a vessel is recorded by GFW as being within the boundaries of the monument. Different vessels are displayed with different colors. Observations are grouped by whether GFW categorized them as fishing events (triangles) or non-fishing events (circles). The dashed vertical line indicates the re-opening of the monument. Days with values of zero are omitted from the graph.

**Figure 13:**
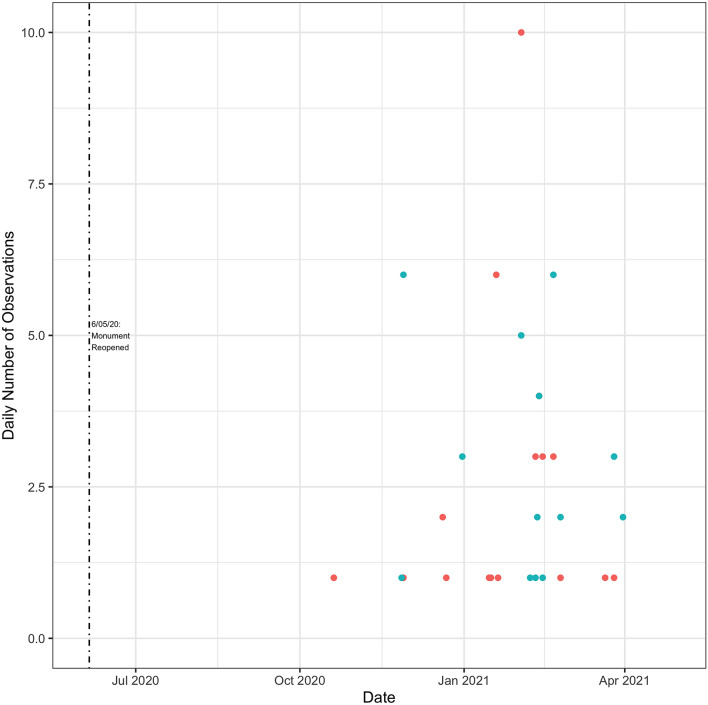
Squid vessel activity inside and near the monument. Blue dots indicate the total number of daily observations inside the monument. Red dots indicate the total number of daily observations outside but near the monument. Near the monument is defined as occurring within a box with bounding coordinates of ($40.6^{\circ }$, $-\,68.268^{\circ }$) and ($38.865^{\circ }$, $-\,65.943^{\circ }$). These coordinates correspond to the most northern, most western, most southern, and most eastern coordinates within the monument itself (coordinates provided by NOAA). Days with values of zero are omitted from the graph.

I now turn to how individual vessels reacted to the reopening of the monument. I focus on tuna vessels (Fig. 10). There is definitely evidence of vessel activity taking place in the monument again but there did not appear to have been a rush to fish in the monument, relative to adjacent areas with similar fishing conditions (Fig. 11). Fishing effort trends inside and outside of the monument were very similar following re-opening. It appears that there was slightly more fishing adjacent to but outside of the monument when the longline fleet first started fishing in this area in late June 2020. I show similar figures for the squid vessels in Figs. 12 and 13. It can be seen that the data is rather sparse and there is no clear pattern, but certainly no evidence of intense fishing pressure inside the monument after it was re-opened.

## Discussion

I conclude that the Northeast Canyons and Seamounts Marine National Monument caused little if any disruption to the squid/butterfish, mackerel, and tuna fisheries. Likewise, reopening the monument provided little tangible economic benefits to these fisheries. I base this conclusion on four main findings. First, following closure of the monument, only a small fraction (less than 1%) of recent historical fishing grounds were closed off. Second, total catch did not decline following the closure of the monument. Pounds landed did not decline for squid, mackerel, and tuna/swordfish in correlation with the fishing prohibition, especially when compared to adjacent regions targeting the same species. Third, none of the potentially impacted fleets were forced to travel further to fish after the monument was closed to fishing. Fourth, following reopening, 99% or more of fishing activity continued to take place outside the monument. The economic arguments made against the monument’s commercial fishing prohibition, including for re-opening the monument to fishing in 2020, do not appear to be supported by the available data.

## Methods

### Data and software

This analysis used two main data sources: (1) annual (through 2020) summaries of landings by species and by region provided by the Atlantic Coastal Cooperative Statistics Program (ACCSP), and (2) vessel-tracking data provided by Global Fishing Watch. The ACCSP is a cooperative state-federal program of U.S. states and the District of Columbia; it was established in 1995 to be the principal source of fisheries-dependent information on the Atlantic Coast of the United States. For the ACCSP data, I obtained annual landings by species for the North Atlantic region, Mid Atlantic region, and South Atlantic region (excluding landings from the Gulf of Mexico). The weekly cumulative landings data was obtained from the NOAA Fisheries Greater Atlantic Quota Monitoring website. Global Fishing Watch is an organization that provides access to information on commercial fishing activities, in particular information on the identity and location of fishing vessels^34^. Many large vessels use a system known as the Automatic Identification System (AIS) to avoid collisions at sea, broadcast their location to port authorities and other vessels, and to view other vessels in their vicinity. Vessels fitted with AIS transceivers can be observed by AIS base stations and by satellites fitted with AIS receivers. The US Coast Guard requires all vessels larger than 65 feet to have an AIS receiver onboard. Global Fishing Watch obtains AIS data for fishing vessels and enables users with Internet access to monitor fishing activity globally, and to view individual vessel tracks. They also partner with academic researchers to provide more fine-scale data.

To obtain the vessel-tracking data for the relevant fisheries, I reviewed NOAA databases of squid and mackerel permits (2019 version; vessels with squid permits are automatically issued a Butterfish permit), and the Atlantic tuna permits (2020 version) and matched each permitted vessel to its unique Maritime Mobile Service Identity (MMSI) number, which is associated with Global Fishing Watch tracking information. I was able to identify 84% (187/224) of squid/butterfish permitted vessels (I focused on the SMB1A (Tier 1) permit category associated with the vast majority ( approx. 99%) of squid catch^35^), 100% of Tier 1 and Tier 2 mackerel-permitted vessels (56/56 vessels), and 74% of active tuna longline vessels (100/135 vessels). “Active” is defined as having reported successfully setting pelagic longline gear at least once between 2006 and 2012^36^. This translates to a total of 17.55 million observations on fishing vessel locations for all three fisheries. I drop any observations that are missing either a latitude or longitude entry. For the squid and mackerel fisheries, I drop any observations with unusual longitudes ($\ge 0^{\circ }$ and $<-\,90^{\circ }$) and latitudes ($\le 30^{\circ }$). For the tuna longline fishery, I drop any observations with a longitude less than $-\,82^{\circ }$ to exclude fishing within the Gulf of Mexico as well as a vessel that takes a trip through the Panama canal. GFW uses a machine learning algorithm to classify each observed vessel location as either engaged in fishing or not engaged in fishing (for example in transit)^37^. I use the same classification and define all recorded fishing locations as fishing activity and classify all recorded locations (fishing and non-fishing) as vessel activity.

**Figure 14:**
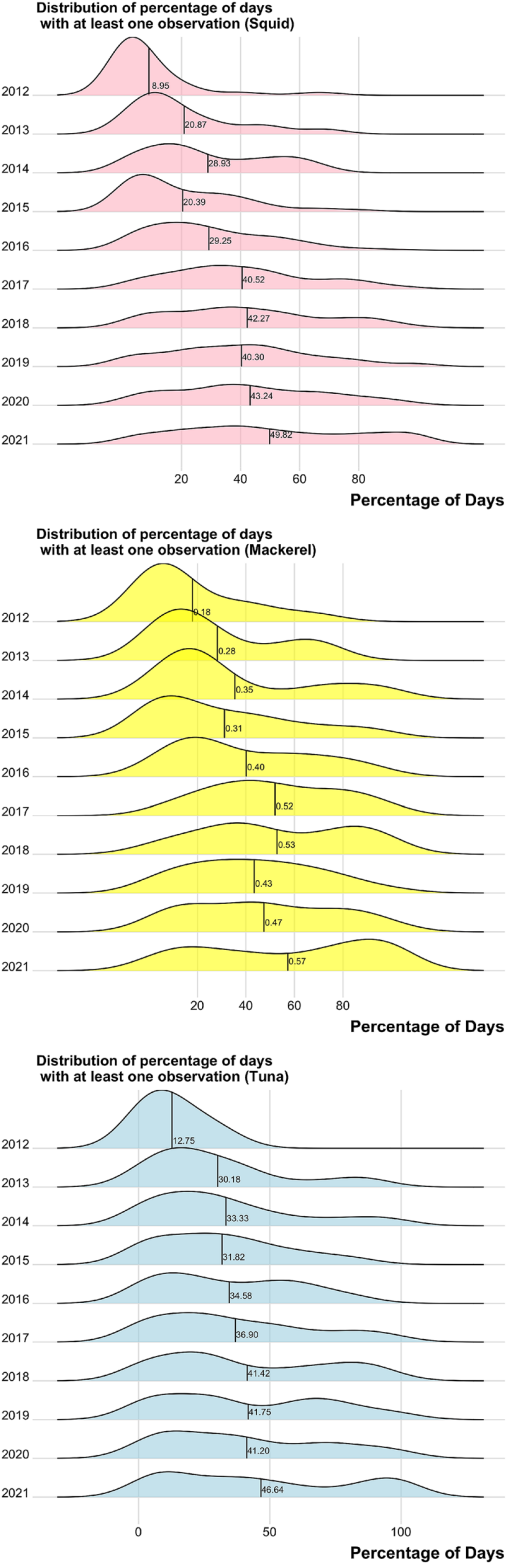
Histograms showing the percentage of days in a year with at least one observation by vessel. Histograms are ordered by permit category and by year, with the mean value highlighted in each year. For example, a mean value of 0.57 implies that the average vessel is observed at least once on 57% of the days in a calendar year. For 2021, the calendar year ends on April 21st, 2021. Figures generated by the author using R version 4.1.1 and RStudio 1.4.1717.

An important question for the GFW data is how extensive is the coverage of the vessels identified. In Fig. 14, I plot a series of histograms showing the percentage of days in a calendar year that a vessel is observed at least once. For 2021, the calendar year ends on April 30th. It can be seen that coverage has improved considerably since 2012 and is now close to 50% for most fisheries. This mean that the location of a vessel is observed, on average, at least once every 2 days. Is this adequate coverage to get a clear picture of vessel activity? It is difficult to answer this question with a high degree of certainty but the answer appears to be yes. For example, in 2018 there was a total of 5623 sets made by vessels in the pelagic longline fishery for Atlantic tuna and swordfish^38^. Considering that there are around 135 active vessels, this corresponds to 42 sets per vessel per year. Therefore a lower bound on days at sea would be around 84 and an upper bound would be around 126. A similar conclusion is reached by looking at information on average trips per year (10) and average days per trip (10): 100 days at sea per year^39^. Since the mean number of days observed in the tuna longline fishery has been above 146 since 2018, this suggests very good coverage of vessel activity. It is more challenging to estimate the degree of daily coverage for squid and mackerel vessels since these vessels are often permitted to operate in multiple fisheries and may operate year-round targeting different species at different times. I do note that the seasons for both species are relatively short (the majority of landings occur within a 5 months window) and mean coverage has been above 40% since 2017 (146 days, just under 5 months).

Finally, the bathymetry data displayed in Fig. 1 was obtained from the GEBCO Compilation Group (2020) GEBCO 2020 Grid (doi:10.5285/a29c5465-b138-234d-e053-6c86abc040b9). All analyses were performed in R version 4.1.1 and RStudio 1.4.1717^40^. All the code necessary to replicate the figures, tables, and analysis in this paper is provided here: https://github.com/lynham/atlantic_monument.

### Blue paradox tests

Near the monument is defined as occurring within a box with bounding coordinates of ($40.6^{\circ }$, $-\,68.268^{\circ }$) and ($38.865^{\circ }$, $-\,65.943^{\circ }$). These coordinates correspond to the most northern, most western, most southern, and most eastern coordinates within the monument itself (coordinates provided by NOAA).

### Distance traveled

To calculate distance traveled, I group vessel locations by vessel and then order them in chronological order. I use the Haversine formula to calculate the distance between all chronological locations. I do not calculate a distance between two locations if the elapsed time between locations is greater than 48 h. The second location is coded as missing for the distance variable (this is typically less than 0.5% of all observations for each fishery). I then sum distance traveled by month by vessel.

### Regression specifications

In Column (1) of Table 2, I estimate a regression of the following form:

1 $$\begin{aligned} {\text{Obs}}_{i,t}=\beta _{0} + \beta _{1}{\text{Inside}}_{i} + \beta _{2}{\text{After}}_{t} + \beta _{3}{\text{Inside}}_{i}*{\text{After}}_{t} + u_{i,t} \end{aligned}$$

where the outcome variable (${\text{Obs}}_{i,t}$) is the total number of observed vessel locations in area *i* on day *t*. ${\text{Inside}}_{i}$ is an indicator variable for whether the observed vessel locations are inside the monument, ${\text{After}}_{t}$ is a time-dependent indicator variable for after the announcement of the monument but before its actual closure (September 15, 2016–November 14, 2016), and ${\text{Inside}}_{i}*{\text{After}}_{t}$ is the two indicator variables interacted with (i.e. multiplied by) each other. $u_{i,t}$ is an unobserved error term. The primary coefficient of interest is $\beta _{3}$, which can be interpreted as the increase in vessel activity inside the monument relative to nearby areas, following the announcement but prior to actual closure. In other words, the Blue Paradox effect.

In Column (2) of Table 2, I estimate a regression of the following form:

2 $$\begin{aligned} {\text{Inside}}_{i,t}=\beta _{0} + \beta _{1}{\text{After}}_{t} + {{\mathbf{v}_{\mathbf{i}}}{\boldsymbol{'\phi }}} + u_{i,t} \end{aligned}$$

where the outcome variable (${\text{Inside}}_{i,t}$) is an indicator variable for whether vessel *i* is inside the monument at time *t*. ${\text{After}}_{t}$ is a time-dependent indicator variable for days after the announcement of the monument but before its actual closure (September 15, 2016–November 14, 2016), **v**_**i**_ is a vector of individual vessel dummies to account for vessel-specific differences in fishing preferences, and $u_{i,t}$ is an unobserved error term. This regression serves as a simple test of whether vessels were more likely to be in the monument after the announcement but prior to its closure.

The main regression equation I estimate in Table 3 is the following:

3 $$\begin{aligned} y_{i,t}=\beta _{0} + \beta _{1}{\text{MON}}_{t} + \beta _{2}{\text{Region}}_{i} + \beta _{3}{\text{Region}}_{i}*{\text{MON}}_{t} + u_{i,t} \end{aligned}$$

where the primary outcome variable ($y_{i,t}$) is the log of landings for region *i* in year *t*. ${\text{MON}}_{t}$ is a dummy variable for the closure of the monument, ${\text{Region}}_{i}$ is a dummy variable indicating whether the landings are for the impacted region, and ${\text{Region}}_{i}*{\text{MON}}_{t}$ is the two dummy variables interacted with (i.e. multiplied by) each other. $u_{i,t}$ is an unobserved error term. The primary coefficient of interest is $\beta _{3}$, which can be interpreted as the effect of the monument closure on landings in the impacted region. Equation 3 is a difference-in-differences equation^41–43^, which implicitly controls for changes in factors such as market forces or fishery management rules that affect both regions. The setup is equivalent to a Before-After-Control-Impact (BACI) design in ecological research using observational data^44,45^.

In Table 4 I estimate regressions of the following form:

4 $$\begin{aligned} y_{i,t}=\beta _{0} + \beta _{1}{\text{MON}}_{t} + {{\mathbf{v}}_{{\mathbf{i}}}{\boldsymbol{'\phi }}} + u_{i,t} \end{aligned}$$

where $y_{i,t}$ is the outcome variable of interest (monthly distance traveled) for vessel *i* in month *t*. $\beta _{0}$ is the standard intercept term and $\beta _{1}$ is the main slope parameter of interest. ${\text{MON}}_{t}$ is a dummy variable that takes the value of 0 for all dates when the monument is open to fishing and 1 for all dates when the monument is closed to fishing. **v**_**i**_ is a vector of individual vessel dummies. The reason for including the vessel dummies is that not all vessels show up in the GFW database prior to the closure of the monument. Thus, the interpretation of $\beta _{1}$ is the average effect of the monument on monthly distance traveled, after controlling for the fact that different vessels tend to have higher or lower monthly averages to begin with.
